# A Systematic Review and Meta-Analysis of Porcine Epidemic Diarrhea Virus Vaccine Efficacy and Its Modifiers

**DOI:** 10.3390/ani15243592

**Published:** 2025-12-14

**Authors:** Shaomei Li, Shuizhu Niu, Bo Yao, Qianlin Chen, Wenjie Ma, Jie Luo, Hua Zheng, Guoyang Xu, Tao Wu, Wei Yao, Liu Yang, Lizhi Fu

**Affiliations:** 1 Chongqing Academy of Animal Science, Chongqing 402460, China; shaomeili123@163.com (S.L.); cecilianiu1999@163.com (S.N.); yaob_chongq@163.com (B.Y.); roger0601@163.com (J.L.); zheng_121_16@163.com (H.Z.); guoyangxu@126.com (G.X.); lll334420@163.com (T.W.); 2 National Center of Technology Innovation for Pigs, Chongqing 402460, China; qlchenjj@163.com (Q.C.); qd1992mwj@163.com (W.M.); 3 Chongqing Engineering Technology Research Center for Veterinary Biological Products, Chongqing 402460, China; 4 Rongchang Field Scientific Observation and Research Station for Animal Epidemics, Ministry of Agriculture and Rural Affairs, Chongqing 402460, China; 5 Animals Husbandry Industry Developnent Center, Wanzhou District, Chongqing 404100, China; yaowei9167@163.com

**Keywords:** porcine epidemic diarrhea, vaccines, vaccine efficacy, vaccine strategies, meta-analysis

## Abstract

Porcine epidemic diarrhea (PED) continues to inflict devastating losses on global swine production. By systematically reviewing and meta-analysing 32 high-quality studies, we quantified the protective efficacy of PED vaccines and dissected how core variables shape that protection. Using fecal scores as the endpoint, we pooled effect sizes under a random-effects model and explored sources of heterogeneity through subgroup analyses. This analysis showed a pooled effect size (Hedges’ g) of −5.80, confirming that vaccination markedly alleviates diarrheal severity. Moreover, substantial heterogeneity was detected across studies. Stratified analyses revealed that vaccine type, vaccination target, vaccination route, and vaccination frequency are the principal sources of variation, all of which significantly influence vaccine efficacy. Conventional inactivated or live-attenuated formulations still dominate the field, but recombinant live-viral platforms are emerging. Piglets and sows represent the most responsive immunological targets. A two-dose regimen consistently outperforms a single injection, and delivery via intranasal, oral or intramuscular routes offers distinct advantages that can be exploited to maximise mucosal and systemic immunity. Overall, the optimal impact on PED prevention and control is achieved when the vaccine is correctly chosen and paired with an appropriate immunization protocol and delivery route.

## Simple Summary

Neonatal piglets infected with porcine epidemic diarrhea virus (PEDV) can suffer mortality rates of up to 100%. Due to the continuous evolution of the PEDV genome, current vaccines provide insufficient cross-protection against circulating field strains and remain limited in safety, immunogenicity, and efficacy against variant strains. Therefore, this study used meta-analysis to systematically evaluate the efficacy of different PEDV vaccines and vaccination strategies for preventing and controlling porcine epidemic diarrhea. The results revealed pronounced differences in vaccine efficacy depending on vaccine type, vaccination frequency, vaccination target, and vaccination route.

## 1. Introduction

Porcine epidemic diarrhea (PED) is a highly contagious acute intestinal infectious disease caused by porcine epidemic diarrhea virus (PEDV) [1]. It was first documented in Europe in 1971 [2]. Since then, it has repeatedly flared regionally and spread globally, causing annual losses of tens of billions of U.S. dollars and exerting an escalating impact on the worldwide swine industry. In neonatal piglets, it manifests as projectile watery diarrhea, persistent vomiting, and metabolic acidosis, with mortality approaching 100% [3,4]. PEDV is a single-stranded, positive-sense RNA virus whose high genetic variability renders existing vaccines insufficiently cross-protective against prevailing field strains [5,6]. A systematic evaluation of the heterogeneous immunogenicity conferred by different PEDV vaccines is therefore essential to delineate their respective advantages and limitations under varying conditions [7], thereby enabling the formulation of more precise immunization strategies.

The PED vaccine market has experienced robust growth in recent years. Currently marketed PEDV vaccines predominantly comprise conventional inactivated vaccines, live-attenuated vaccines, and genetically engineered vaccines derived from the classical GⅠ-type strain CV777 [8,9]. According to the Global Market Report, it is projected to expand from $1.67 billion in 2024 to $1.80 billion in 2025, representing a compound annual growth rate (CAGR) of 7.7%. In practical applications, inactivated vaccines offer distinct advantages, including an excellent safety profile, high thermostability, and scalability in production [10]. Nevertheless, inactivated vaccines elicit comparatively weak mucosal immune responses, and their immunogenicity is highly susceptible to dose and booster regimens. Moreover, neither classical nor contemporary field variants are effectively or consistently protected against [11,12]. Live-attenuated vaccines, by contrast, possess superior immunogenicity and efficiently induce mucosal immunity. Wang et al. [5] reported that PEDV live-attenuated vaccine strains can recombine with field strains, thereby generating novel, highly virulent variants, indicating that they pose safety risks in clinical applications.

Genetically engineered vaccines, ranging from subunit and recombinant viral-vectored to nucleic-acid platforms, have been progressively introduced for PED control [13,14,15,16,17]. Subunit formulations, which exclude extraneous viral structural proteins, are characterized by high safety margins, elevated antigenic titres, and favourable cold-chain logistics [18]. However, their immunogenicity is profoundly influenced by the vaccination route and the specific antigens selected [19]. Yu et al. [20] demonstrated that piglets receiving two intramuscular doses of a PEDV subunit vaccine at 2-week intervals elicited neutralising antibodies, yet still developed severe diarrhoea following virulent challenge at five weeks of age. Recombinant viral-vectored and nucleic-acid vaccines remain encumbered by unresolved concerns regarding biosafety and genetic stability [21,22], necessitating further exploration of their immunization routes and strategies.

Meta-analysis, as a statistical method for quantitatively integrating data from independent studies, can not only systematically assess the overall effect of vaccine immunization through effect size pooling, heterogeneity testing, and subgroup analysis, but also identify sources of variation across studies [23]. In this study, we conducted a meta-analysis of peer-reviewed publications on PEDV immunisation to systematically appraise vaccine efficacy. Subgroup analyses were further employed to dissect the contributions of vaccine category, booster frequency, vaccination target, and administration route to immune outcomes, thereby pinpointing key determinants and clarifying the origins of heterogeneity. The resultant evidence base is intended to inform the optimisation of PED immunisation and prevention strategies.

## 2. Materials and Methods

### 2.1. Search Strategy

The authors performed a non-registered systematic review independently and followed the Preferred Reporting Items for Systematic Reviews and Meta-Analyses (PRISMA) 2020 statement [24]. We performed the PRISMA 2020 checklist (Table S1) to ensure the relevant information included was consistent with the study criteria. The studies were written in either English or Chinese, and they were identified using PubMed (https://pubmed.ncbi.nlm.nih.gov/) for English studies and CNKI (https://www.cnki.net/) for Chinese literature. The search was conducted on 1 January 2025, covering all publications from inception to that date. The terms “Porcine epidemic diarrhea”, “PED”, “Porcine Epidemic Diarrhea Virus”, “PEDV”, “vaccine”, “immunogenicity”, and “fecal score” were used as the keywords for advanced search, and they were set to be used as synonyms for the purpose of expanding the search scope. Subsequently, the compilation was performed by two authors, the duplicate records were removed, and the relevance of the results was analyzed (firstly scanning the title and abstract, and if applicable the full text). Manuscripts out of scope were removed in this phase. The following inclusion criteria were used to select articles. All records were imported into EndNote X9, and duplicates were removed automatically and manually verified.

### 2.2. Inclusion and Exclusion Criteria

The inclusion criteria were as follows:

(1) Studies in which swine vaccines are used for the prevention of epidemic diarrhea
(2) The primary outcome must be included the fecal score
(3) The research must include both a vaccine-immunized group and a control group
(4) The study data must report the following for fecal score: Mean value ($\bar{x}$), Sample size (*n*), Standard deviation (SD) or Standard error (SE).

The exclusion criteria were as follows:

(1) Studies exclusively using mice as animal models
(2) conference abstracts, letters to the editor, and reviews
(3) Literature with incomplete data or insufficient information for valid extraction

### 2.3. Database Formation

This study incorporated 54 datasets from 32 eligible literatures into the Meta-analysis, summarizing the following data information: mean ($\bar{x}$), sample size (*n*), and standard deviation (*s*) of fecal scores for the vaccinated treatment group (t) versus the unvaccinated control group (c). For literatures providing only standard error (*se*), *s* was calculated using the formula: $s=se\sqrt{n}\left(1\right)$. If literatures did not report *s* or *se*, *s* was assigned as 1/10 of the mean value [25]. Data were extracted independently by two investigators (Shuizhu Niu and Bo Yao) and checked by other authors. Data from figures were extracted using GetData Graph Digitizer software (version 2.26). From all included publications, we extracted the following information: first author, year of publication, vaccine type, vaccination frequency, vaccination target, and vaccination methods.

### 2.4. Statistical Analysis

The meta-analysis was performed in RStudio (version 2025.05.1) using the meta package [26]. The Standardized Mean Difference (SMD), adjusted by Hedges’ g correction, was used as the primary effect size metric. The absolute value of the SMD reflects the magnitude of vaccine efficacy in reducing diarrheal incidence in swine populations, categorized as follows: |SMD| < 0.3 (Small effect), 0.3 ≤ |SMD| < 0.8 (Medium effect), |SMD| ≥ 0.8 (Large effect). Heterogeneity among studies was assessed using Cochran’s *Q* test and I^2^ statistic. A random-effects model was applied when significant heterogeneity was indicated (*Q*-test *p*-value < 0.05 or I^2^ > 50%); otherwise, a fixed-effects model was used. To explore potential sources of heterogeneity, subgroup analyses were conducted using moderator variables (Table 1). Robustness of the findings was evaluated through sensitivity analysis using Rosenberg’s fail-safe number (NF). Publication bias was considered unlikely if NF exceeded 5*k* + 10 (*k* = number of included studies).

## 3. Results

### 3.1. Study Selection and Data Extraction

**Figure 1:**
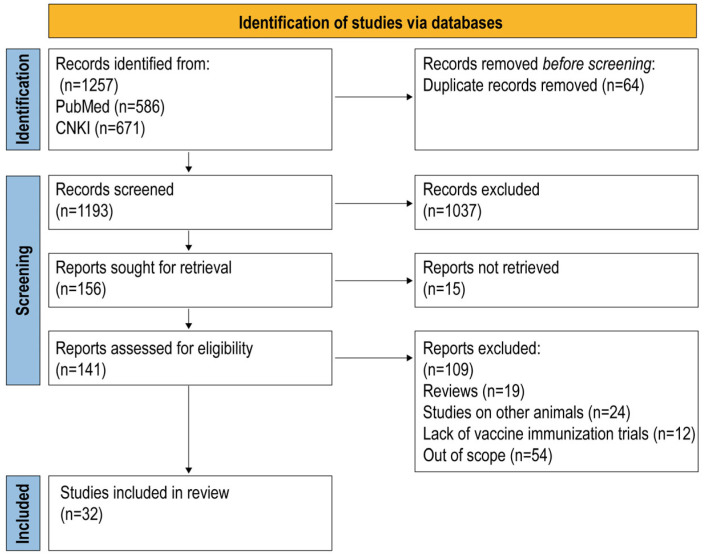
PRISMA flow diagram of the systematic review from the initial search and screening to the final selection of publications included in the study.

Due to heterogeneous sample types and methodologies employed across studies for antibody titer determination, this endpoint was not incorporated into the meta-analysis. Moreover, mortality data were excluded because of insufficient statistical power. Consequently, fecal score was selected as the primary outcome to evaluate PEDV vaccine efficacy in the present study. The search of two databases identified 1257 records. After removing duplicate studies and preliminary screening, 1193 papers remained. Title and abstract screening reduced the pool to 156 potentially relevant papers. We were unable to evaluate the full text of 15 articles. After reading the full text, a total of 109 articles were excluded for the following reasons: review articles, studies used mice for testing, papers performed pig experiments without a challenge and out of scope for analysis. Ultimately, a meta-analysis of 32 publications (54 studies) was performed (Figure 1). The fecal status after challenge was scored on a 4-point scale from 0 to 3 (0; normal and no diarrhea, 1; mild and fluidic feces, 2; moderate watery diarrhea, 3; severe watery and projectile diarrhea) in 21 publications. Additionally, 8 publications were scored on a 5-point scale of 0 to 4, and 3 publications on a 3-point scale of 0 to 2.

### 3.2. Meta-Analysis of PEDV Vaccines Efficacy

**Figure 2:**
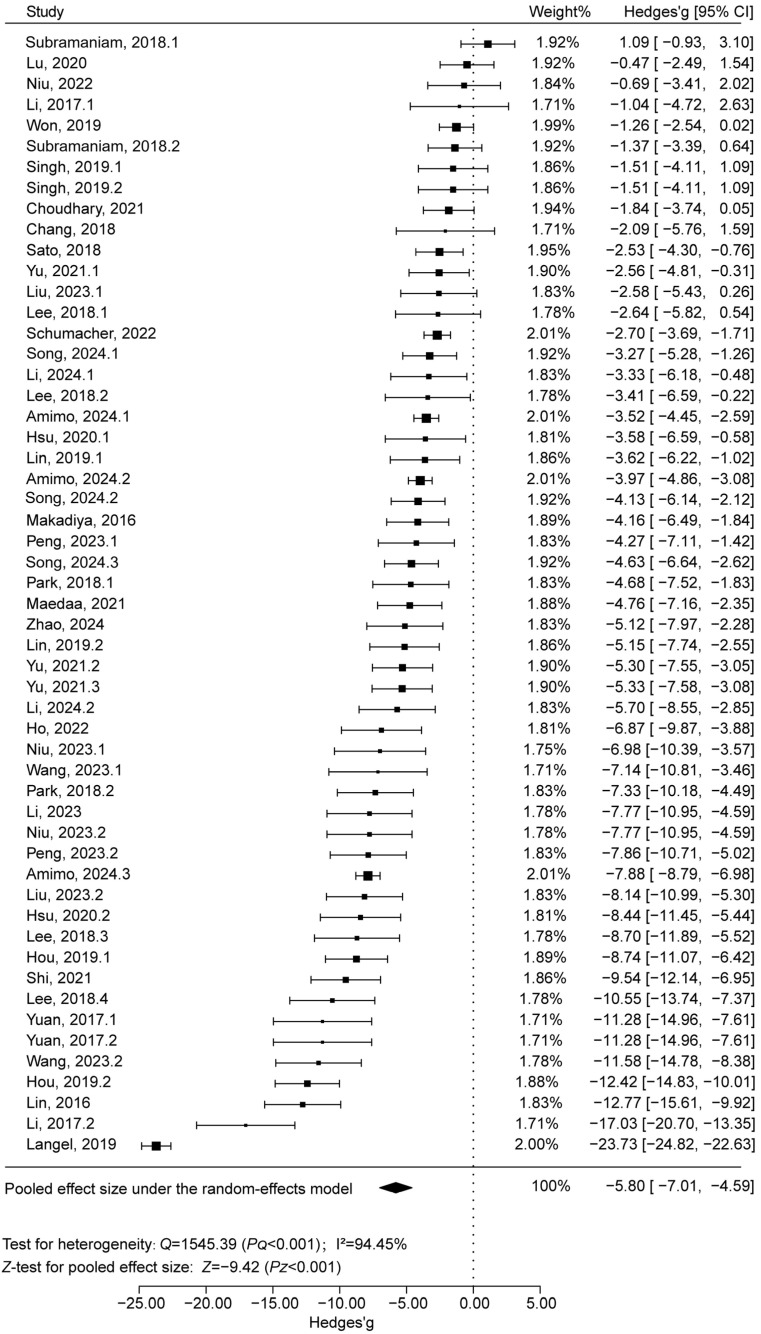
Forest plot of PEDV vaccine effectiveness based on fecal score. The first column “Study” included “Author + Year” [15,20,27,28,29,30,31,32,33,34,35,36,37,38,39,40,41,42,43,44,45,46,47,48,49,50,51,52,53,54,55,56,57]. Point size reflects the relative weighting of the study to the overall effect size estimated, where a larger point size represents a greater weight and the combined effect size estimated, including the confidence intervals. The diamond represents the overall effect. Weight: represented as a percentage and indicates the influence of the study on the overall result; CI: confidence interval.

The analysis of post-challenge fecal scores revealed significant heterogeneity among the 54 included studies (*Q* = 1545.39, df = 53, *p* < 0.001; I^2^ = 94.45%). A random-effects model was used to pool the effect sizes, yielding a Hedges’ g of −5.80 (*Z* = −9.42, *p* < 0.001), which indicates a strong protective effect. This confirms that vaccination is an effective strategy for reducing diarrhea in pigs. In addition, publication-bias testing yielded a fail-safe number (N) of 1070, exceeding 5*k* + 10 = 280 (where *k* is the number of studies), further supporting the reliability of the meta-analysis results (Figure 2).

### 3.3. Heterogeneity Assessment

Heterogeneity tests for four moderators (vaccine type, vaccination target, vaccination frequency, and vaccination route) showed that each contributed significantly to the observed heterogeneity (*Q*_M_ tests, all *p* < 0.001; Table 2). Consequently, subgroup analyses were warranted.

### 3.4. Subgroup Analysis

#### 3.4.1. Effect Analysis Based on Vaccine Type

**Figure 3:**
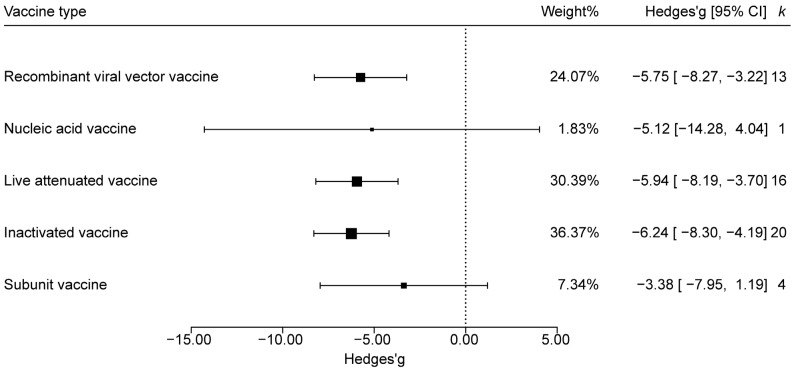
Effects of different vaccine types on the efficacy of PEDV vaccine. Point size reflects the relative weighting of the study to the overall effect size estimated, where a larger point size represents a greater weight and the combined effect size estimated, including the confidence intervals. Weight: represented as a percentage and indicates the influence of the study on the overall result; CI: confidence interval; *k*: sample size of the influencing factors. The same conventions apply to the subsequent figures.

The vaccine type of the PEDV vaccines used in this analysis was classified into recombinant viral vector vaccines, nucleic acid vaccines, live attenuated vaccines, inactivated vaccines, and subunit vaccines. Forest plots based on fecal scores showed that the 95% CIs for nucleic-acid and subunit vaccines crossed the null line (Hedges’ g = 0), indicating no significant protection. In contrast, the CIs for recombinant viral-vector, live-attenuated, and inactivated vaccines lay entirely below zero, denoting significant protective effects. Ranked by absolute weighted effect size, inactivated vaccines were most effective, followed by live-attenuated and then recombinant viral-vector vaccines (Figure 3).

#### 3.4.2. Effect Analysis by Vaccination Target

**Figure 4:**
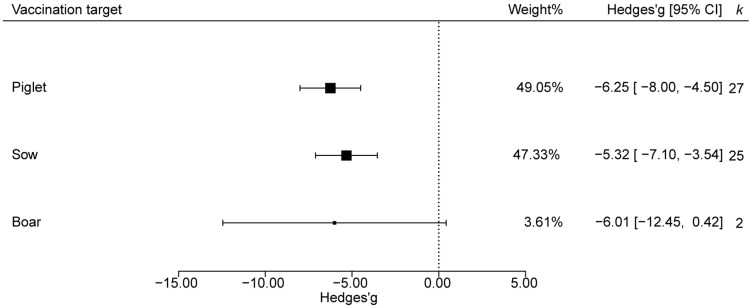
Impact of vaccination target on PEDV vaccine efficacy.

Vaccination targets were categorized as sows, piglets, or boars. Fecal-score analysis revealed no significant protection in boars, whereas both piglet and sow groups showed significant efficacy. The absolute effect size and statistical weight were larger in piglets than in sows, indicating that vaccinating piglets induced the strongest immune response (Figure 4).

#### 3.4.3. Effect Analysis Based on Vaccination Frequency

**Figure 5:**
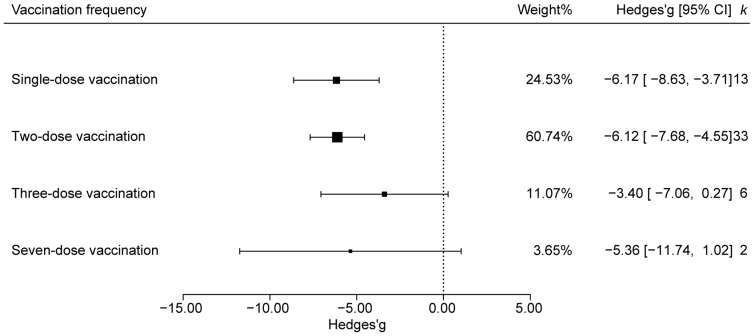
Effects of different vaccination frequencies on the efficacy of PEDV vaccine.

Vaccination frequencies were grouped as single, two, three, or seven doses vaccination. Based on the analysis of the fecal-score data, the confidence intervals of the effect sizes for the three-dose and seven-dose vaccination groups intersected with the null line. In contrast, both the single and two-dose regimens produced effect sizes whose CIs lay entirely below zero, denoting significant efficacy. Although the absolute effect sizes were similar, the two-dose regimen carried greater statistical weight (Figure 5).

#### 3.4.4. Effect Analysis by Vaccination Route

**Figure 6:**
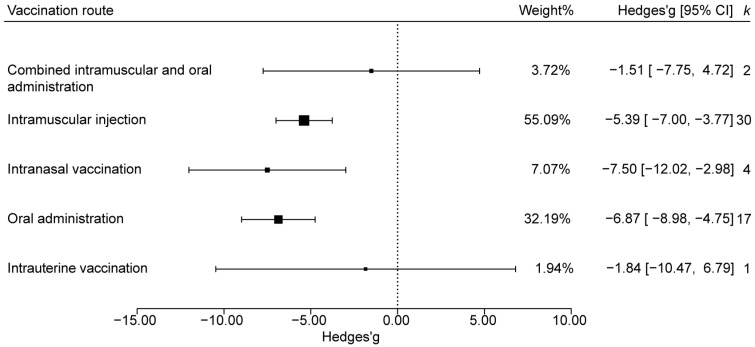
Effects of different vaccination routes on the efficacy of PEDV vaccine.

The vaccination routes of PEDV vaccines were classified into intramuscular injection (IM), oral administration (PO), intranasal vaccination (IN), intrauterine vaccination (IU), or IM combined with PO. Based on the analysis of the fecal score data, the confidence interval of the effect size for the intrauterine vaccination combined with IM and PO group intersected with the line of no effect, indicating no significant protection. In contrast, IN, PO, and IM alone each produced effect sizes significantly below zero, denoting significant protective effects (Figure 6). Ranked by absolute effect size, IN were most effective, followed by PO and then IM; ranked by statistical weight, IM were most effective, followed by PO and then IN.

## 4. Discussion

**Figure 7:**
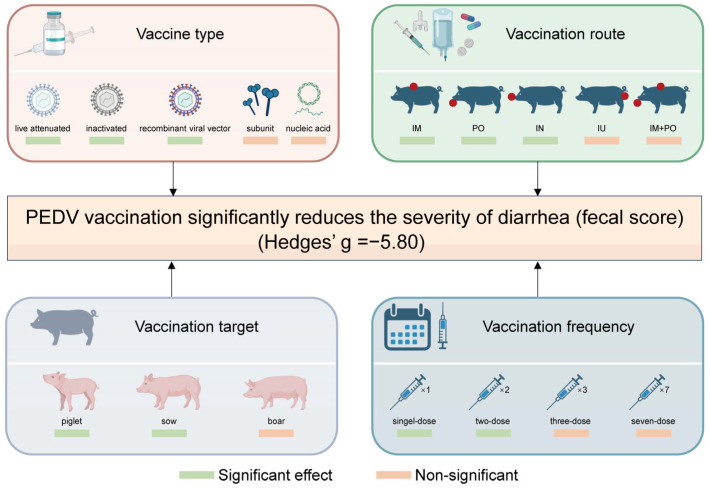
Key Determinants of PEDV Vaccine Efficacy. IM, intramuscular; IN, intranasal; PO, oral; IU, intrauterine.

Preventing and controlling PED remains a major challenge for the swine industry [58]. Vaccination, as the primary intervention, requires accurate evaluation and continuous optimization of its efficacy for clinical application. By integrating 32 independent studies comprising 54 data sets, this meta-analysis confirmed a large pooled effect size for PEDV vaccines and documented that vaccine type, vaccination target, vaccination frequency, and vaccination route are the main sources of heterogeneity (Figure 7).

This study found that conventional inactivated, live-attenuated, and recombinant live-viral vaccines markedly influenced immunogenicity, whereas nucleic-acid and subunit vaccines did not. The discrepancy may arise not only from unequal sample sizes, but also from intrinsic compositional differences. Subunit and nucleic-acid vaccines remain under-represented in the literature; the former present isolated antigens that are inherently less immunogenic than vaccines containing intact pathogens [59,60], while the latter still face the challenge of achieving consistently high immunogenicity [61,62]. Notably, the nucleic-acid vaccine subgroup comprised only one study (*k* = 1), yielding severely inadequate statistical power. The observed lack of significant efficacy (*p* > 0.05) most likely reflects this sample-size constraint rather than a true absence of effect. Most commercially available PEDV vaccines are inactivated or attenuated products based on GⅠ/GⅡ strains, with reported efficacies ≥ 80% [63,64,65]. Subunit vaccines account for only a minor fraction, and no licensed nucleic-acid or recombinant live-viral vaccines are yet on the market. Therefore, to counter the increasing diversity of PEDV strains, it is imperative both to improve the efficacy and safety of traditional vaccines for routine prophylaxis and emergency use, and to intensify the development of next-generation platforms that can enhance overall PED control.

Our results reveal significant heterogeneity in vaccine efficacy among piglets, sows, and boars, a disparity that likely mirrors the biological diversity of immune responses at different physiological stages and in distinct populations. Secretory IgA (SIgA) delivered via colostrum is the principal source of passive immunity for neonatal piglets, and its titre correlates positively with the antigenic match between vaccine and field strain [66,67,68]. The large effect observed in piglets probably reflects both the dynamic regulation of their mucosal immune system and the efficiency of maternal antibody transfer. Studies have demonstrated that the S mRNA-LNP vaccine not only confers active protection against PEDV in immunized piglets, but also provides efficient passive immunity to neonates via colostrum-derived antibodies following sow vaccination [38]. Additional investigations have confirmed that a PEDV S-protein subunit vaccine elicits significantly higher IgG and neutralizing antibody titers in both sows and piglets, and enables highly efficient passive transfer of maternal antibodies to newborn piglets through colostrum [69]. Most clinical trials to date have concentrated on sow herds, whereas the limited number of boars enrolled reduces statistical power and widens confidence intervals.

Our analysis revealed a non-linear relationship between the number of vaccinations and effect size. A single dose and two doses both produced significant and virtually identical pooled effects, whereas the 95% confidence intervals for three or seven doses crossed the null line. This observation may reflect the mucosal immune system’s homeostatic response to repetitive antigenic challenge: excessive boosting can overload the immune network or trigger stress responses that ultimately blunt protection. Alternatively, the plateau could be explained by original antigenic sin or the induction of tolerance. Studies on SARS-CoV-2 (another member of the Coronaviridae) show that repeated administration of inactivated vaccines consolidates a robust response to the ancestral strain while attenuating reactivity to emerging variants [70]. In field practice, a two-dose priming schedule is therefore standard. The first shot is usually live-attenuated, followed by an inactivated vaccine, or two live doses plus one inactivated boost. Future work must dissect the mechanistic links between boosting frequency and vaccine efficacy to refine these protocols.

Vaccination route significantly modulated vaccine efficacy. IN delivery yielded the largest absolute effect size yet contributed only 7.07% of the pooled weight due to the limited number of studies and small sample sizes. Compared with PO, IN elicits higher titres of neutralising antibodies in the intestinal lumen of piglets, indicating superior induction of gut mucosal immunity [70]. However, technical complexity and reproducibility concerns may limit its practical adoption. Although IM produced a marginally lower effect size, its operational standardisation and ease of implementation preserve its dominance in current production systems. IM combined with PO theoretically offers broader protection but did not reach statistical significance in the present analysis, probably constrained by vaccine-intrinsic properties and inter-individual variability. Elucidating the synergistic mechanisms underlying these divergent delivery routes therefore warrants further investigation.

This study has several limitations. Since our study analyzed papers that were written and published in English and Chinese, excluding research papers published in languages other than English and Chinese from this analysis can be considered a limitation. In addition, using only the fecal scores for the analysis as well as failing to analyze the mortality and antibody titers in the serum after challenge were other limitations.

## 5. Conclusions

Vaccination remains the cornerstone of PED control. The meta-analysis confirms that inactivated vaccines, combining a large, pooled effect size with well-established manufacturing protocols, should be the primary choice at present. Sows and piglets must be the central targets of any immunisation programme, and the optimal window for vaccination should be defined by the kinetics of maternally derived antibody decay. A single-dose or two-dose regimen secures robust protection while minimising cost, and exact schedules must be tailored to farm-specific conditions. Although IN delivery shows clear promise, formulation improvements are required to guarantee stability and ease of use. Future work should therefore not only explore novel vaccine platforms and high-efficacy delivery routes, but also refine antigen design and timing of administration, thereby fully exploiting the potential of safe, effective, and user-friendly vaccines against PED.

## Figures and Tables

**Table 1:** Moderator Variables and Sample Sizes Across Included Studies.

Moderator Variables	Type	*k*
vaccine type	Recombinant viral vector vaccine	13
	Nucleic acid vaccine	1
	Live attenuated vaccine	16
	Inactivated vaccine	20
	Subunit vaccine	4
vaccination target	Piglet	27
	Sow	25
	Boar	2
vaccination route	Intranasal vaccination	4
	Intrauterine vaccination	1
	Oral administration	17
	Intramuscular injection	30
	Combined intramuscular and oral administration	2
vaccination frequency	single-dose vaccination	13
	two-dose vaccination	33
	three-dose vaccination	6
	seven-dose vaccination	2

*k*: number of included studies.

**Table 2:** Heterogeneity test of factors.

Influencing Factor	*k*	*Q*_M_	*PQ*_M_
vaccine type	54	85.63	<0.001
vaccination target	54	86.90	<0.001
vaccination frequency	54	88.80	<0.001
vaccination route	54	93.87	<0.001

*k*: sample size of the influencing factors; *Q*_M_: heterogeneity *Q* value for the influencing factors; *PQ*_M_: significance test value for *Q*_M_.

## Data Availability

Data supporting the reported results are contained within the article.

## Supplementary Materials

The following supporting information can be downloaded at: https://www.mdpi.com/article/10.3390/ani15243592/s1, Table S1: PRISMA 2020 checklist.
